# Influence of *ABCB1* Gene Polymorphism on Rivaroxaban Blood Concentration and Hemorrhagic Events in Patients With Atrial Fibrillation

**DOI:** 10.3389/fphar.2021.639854

**Published:** 2021-04-14

**Authors:** Yan Wang, Min Chen, Hui Chen, Fang Wang

**Affiliations:** ^1^ Department of Cardiovascular Medicine, Beijing Hospital, National Center of Gerontology, Institute of Geriatric Medicine, Chinese Academy of Medical Sciences, Beijing, China; ^2^ Laboratory of Cardiology, Beijing Hospital, National Center of Gerontology, Beijing, China; ^3^ Department of Pharmacy, Fujian Provincial Hospital, Fuzhou, China; ^4^ Fujian Provincial Clinical College of Fujian Medical University, Fuzhou, China; ^5^ Department of Cardiology, Fujian Provincial Clinical College of Fujian Medical University, Fuzhou, China

**Keywords:** atrial fibrillation, non-vitamin K anticoagulants, rivaroxaban, ABCB1, gene, pharmacogenetics

## Abstract

**Background and Objectives:** Genetic data on the pharmacokinetics of rivaroxaban and identification of factors that affect its biotransformation, distribution, and excretion will allow for generation of algorithms for personalized use of this drug in patients with atrial fibrillation (AF). Here we tested the effects of *ABCB1* (ATP-binding cassette subfamily B member 1) polymorphisms on the valley rivaroxaban blood concentration and on the frequency of hemorrhagic events in patients with AF and propose a personal anticoagulation therapy management protocol.

**Patients and Methods:** This is a retrospective study. We enrolled Mongolian descent patients who met the criteria from May 2018 to August 2019 in Beijing and Fujian. Clinical data on gender, height, weight, liver and kidney functions, drug trough concentration, and drug dosage were collected; we recorded the bleeding events until 6 months after initiating the medication. *ABCB1* single nucleotide polymorphisms including rs1128503, rs1045642, and rs4148738 were identified. After reaching the steady state of plasma concentration, the peripheral blood was collected to detect the trough rivaroxaban plasma concentrations before the next medication.

**Results:** We included 155 patients in this study including 81 men and 74 women, with an average age of 71.98 ± 10.72 years. The distribution of *ABCB1* genotypes conformed to the Hardy–Weinberg equilibrium. Multiple comparisons between wild (TT) and mutant (CT and CC) genotypes at the rs1045642 locus showed no significant differences of rivaroxaban trough concentrations (TT vs. CT, *p* = 0.586; TT vs. CC, *p* = 0.802; and CT vs. CC, *p* = 0.702). Multiple comparison between wild (TT) and mutant (CC) genotypes at the rs1128503 locus revealed a significant difference of rivaroxaban trough concentrations (TT vs. CC, *p* = 0.0421). But wild (TT) vs mutant (CT) genotypes and mutant CT vs mutant CC genotypes at the rs1128503 locus showed no significant differences of rivaroxaban trough concentrations (TT vs. CT, *p* = 0.0651; TT vs. CT, *p* = 0.6127). Multiple comparisons between wild (GG) and mutant (AG and AA) genotypes at the rs4148738 locus showed no significant differences of rivaroxaban trough concentrations (GG vs. AG, *p* = 0.341; GG vs. AA, *p* = 0.612; AG vs. AA, *p* = 0.649). There was no significant correlation between ABCB1 gene variation loci rs1045642, rs1128503, rs4148738 and bleeding events.

**Conclusion:** rs1128503 locus variations are correlated with the serum concentration of rivaroxaban in patients of Mongolian descent. But no significant correlation between rs1128503 locus variations and bleeding events were obtained.

## Introduction

Bleeding events used to be common occurrences due to hunting traumas and wartime, but embolisms have become more frequent medical emergencies. Acute coronary syndromes, atrial fibrillation (AF), vein thrombosis, and pulmonary embolism all call for anticoagulation therapy. Thromboembolic events are a significant source of mortality and morbidity and for some patients such as those with AF require life-long anticoagulation therapy (Mackman, 2008).

Warfarin has been the main oral anticoagulant in use since its discovery in 1954 by the Wisconsin Alumina Research Foundation (Pirmohamed, 2006). Its action mechanisms include vitamin K epoxide reductase inhibition; coagulation factors II, VII, IX, and X attenuation; and protein C and S inhibition (Mekaj et al., 2015). Warfarin helps prevent strokes in patients with AF and is the most important anticoagulant agent, but it also has many limitations including the need for frequent laboratory monitoring, unwanted drug and food interactions, slow onset of action, and a narrow therapeutic window (Lip and Agnelli, 2014). Thus, new direct oral anticoagulants (DOACs) such as dabigatran, rivaroxaban, apixaban, and edoxaban have been studied and manufactured to try to meet the needs of clinical anticoagulation without the shortcomings of warfarin (Thachil, 2014; Michalcová et al., 2016; Burn and Pirmohamed, 2018; Franco Moreno et al., 2018). DOACs act specifically on a single target (either thrombin or factor Xa) to inhibit clot formation and fibrin deposition (Lip and Agnelli, 2014). Rivaroxaban is a reversible factor 10a inhibitor, and its usage has been increasing rapidly. Its advantages include the elimination of the need for coagulation monitoring, a rapid onset of action, less of food interactions, fewer drug interactions, and a wide therapeutic window (Lip and Agnelli, 2014; Mekaj et al., 2015; Cherubini et al., 2018). Rivaroxaban can be convenient for patients since it is administered in fixed dosage schedules (Bauer, 2013).

Excretion of rivaroxaban occurs through two main pathways: cytochrome P450 CYP2J2-and CYP3A4-dependent metabolisms are responsible for two-thirds of its elimination, whereas the rest (one-third) is excreted renally and unchanged (Eriksson et al., 2009; Pfeilschifter et al., 2013; Yates and Sarode, 2013). Rivaroxaban is a substrate of the p-glycoprotein (P-gp) efflux transporter (Gnoth et al., 2011) encoded by the *ABCB1* gene.

Although DOACs have predictable pharmacokinetics and pharmacodynamics, and do not require routine coagulation monitoring, it has recently been reported that there are significant differences in plasma and drug reactions among individuals (Hoffmeyer et al., 2000; Ing Lorenzini et al., 2016). Although several factors such as age, race, gender, smoking, and diet can lead to inter-individual variability of DOACs (Sweezy and Mousa, 2014). But these factors are not enough to explain the current clinical confusions. Anticoagulants’ potencies vary among different patients, and increasing the efficacy and safety of anticoagulation is essential. (Paré et al., 2013; Sweezy and Mousa, 2014; Dimatteo et al., 2016).

In clinical practice, we also found the same problem. When using DOACs in clinical, some strange phenomena began to perplex us doctors. For example, when rivaroxaban was used normally, the plasma concentration of rivaroxaban could be distributed in the range of <20 to >400 ng/ml, and the valley range and peak range were quite large, even overlapping areas appeared. In addition, with the emergence of some unexplained drug safety events, doctors began to suspect that extremely low valley concentration and extremely high peak concentration are likely to make some patients face the risk of thrombosis or bleeding. This situation often means risk for acute or critical patients.

In our clinical work, a 30-year-old young woman in Fuzhou developed cerebral hemorrhage after taking a tablet of rivaroxaban. A 42 years old chef took rivaroxaban 20 mg once a day for 1 week. After that, massive subcutaneous congestion appeared, and his hemoglobin level decreased from 126 to 82 g/L. The plasma concentration of rivaroxaban was still very high (320 ng/ml) even if it was 3 days after the withdrawal of rivaroxaban. These phenomena perplex us and the known influencing factors can not explain them.

Genetic polymorphisms are thought to contribute to the wide intraindividual variability seen in anticoagulant drug responses. For rivaroxaban, the principal drug interactions are mediated by CYP3A4 and P-gp (Kim et al., 1999; Eriksson et al., 2009; Gnoth et al., 2011; Mueck et al., 2013). P-glycoprotein (P-gp), also known as multidrug resistance protein (MDR), is one of the main members of ABC (ATP binding cassette) transporter superfamily. It is the first drug transporter found related to multidrug resistance. P-glycoprotein was first found in tumor cells, and later found in a variety of tissues, such as liver, kidney, intestine, placenta, blood brain barrier, blood testis barrier, hematopoietic stem cells, peripheral blood monocytes, mature macrophages, natural killer cells and lymphocytes. P-glycoprotein plays a key role in the physiological defense system, also plays a role in the absorption, distribution and metabolism of drugs, so it has been widely concerned. ABCB1 gene polymorphisms cause P-glycoprotein expression to increase or decrease, which may affect the absorption, distribution and metabolism of drugs in the body, resulting in individual differences in drug effects.

*ABCB1* has more than 100 polymorphisms, among which rs2032582 and rs1045642 have been proved to affect the metabolism of rivaroxaban (Ing Lorenzini et al., 2016; Gouin-Thibault et al., 2017; Asic et al., 2018). The SNPs of *ABCB1* were significantly different in different races (Ing Lorenzini et al., 2016). There are few studies on the polymorphism of *ABCB1* gene encoding P-gp, and only a few clinical studies have evaluated whether they explain the difference in pharmacokinetics of rivaroxaban, but no positive association was found in Caucasian populations (Asic et al., 2018).

The confusion in clinical work and too few studies at present made us pay attention to this content, so we conducted a prospective study to find the gene loci that affect the metabolism of rivaroxaban and propose a personal anticoagulation therapy management protocol. We collected clinical data such as gender, height, weight, liver, kidney functions, drug trough concentration, drug dosage, and we recorded bleeding events until 6 months after initiating the medication. This article is part of the study, we did a retrospective analysis of some of the patients we enrolled. We reviewed 155 patients enrolled in the research to analyze the influence of *ABCB1* gene polymorphism on rivaroxaban blood concentration and hemorrhagic events in patients with atrial fibrillation. The characterization of bleeding events in this study was consistent with the ROCKET-AF and RE-LY trial bleeding events definition (Connolly et al., 2009).

Here, we assessed the effects of *ABCB1* polymorphisms on the valley serum rivaroxaban concentration and on the frequency of hemorrhagic events in patients with AF and propose a personal management protocol for anticoagulation therapy.

## Patients and Methods

### Patients

One hundred fifty-five Mongolian race patients diagnosed as having nonvalvular AF and initiated on anticoagulation therapy admitted to Beijing Hospital and Fujian Provincial Hospital from May 2018 to August 2019 were enrolled in this study. All patients enrolled were prescribed rivaroxaban (Xarelto; Bayer Pharma AG, Berlin, Germany) once daily. Adherence was confirmed. After reaching the steady state of plasma concentration, blood collection for trough plasma concentration measurement was performed 24 h after drug administration (drug concentration valley value). These blood samples were stored at −20°C until analysis. The dose of rivaroxaban (20 or 15 mg once daily) was determined by doctors. Estimated glomerular filtration rate (eGFR) were calculated using the Cockroft–Gault equation.

In addition, we collected clinical data such as gender, height, weight, liver, kidney functions, drug trough concentration, drug dosage, and we recorded bleeding events until 6 months after initiating the medication.

The study protocol was approved by the Hospital Ethics Committees of the Beijing Hospital (project identification code: 2016BJYYEC-040-02) and Fujian Provincial Hospital (project identification code: K2017-10-005). All the patients in the study signed a voluntary informed consent; their data were recorded in the impersonal patient cards.

The main inclusion criteria are: 1) age older than 18 years; 2) confirmed diagnoses of nonvalvular AF and need of anticoagulation therapy (absence of artificial heart valves and hemodynamically significant mitral stenosis). Attending physicians made the diagnoses on the clinical features and on results of examinations (electrocardiogram for AF); 3) Willing and able to provide written informed consent.

We excluded patients with confirmed diagnosis of valvular AF (presence of artificial heart valves and hemodynamically significant mitral stenosis), high risk of bleeding, stroke within 1 month or any history of hemorrhagic or lacunar stroke, known non-cardiovascular disease that is associated with poor prognosis, those with creatinine clearance ≤15 ml/min, abnormal liver function >2 times the normal value, any known hepatic disease associated with coagulopathy and hyperthyroidism, active infective endocarditis, hypertrophic obstructive cardiomyopathy, dementia, and impaired swallowing, and those simultaneously using DOACs and drug groups such as CYP3A4 and P-gp inhibitors (amiodarone, verapamil, diltiazem, quinidine, ticagrelor, and clarithromycin), CYP3A4 and P-gp inducers (rifampicin, carbamazepine, phenobarbital, and phenytoin, pantoprazole, and atenolol).

We also excluded patients with history of hypersensitivity or known contraindication for rivaroxaban, subjects who are pregnant, breastfeeding, or are of childbearing potential, previous assignment to treatment during this study, concomitant participation in another study with investigational drug, known contraindication to any study-related procedures. At the end, we enrolled 155 patients with AF: 81 men and 74 women, with a mean age of 71.98 ± 10.72 years.

### Sample Collection and Determination of Rivaroxaban Trough Plasma Concentrations

After reaching the steady state of blood concentration (after five half-lives), we collected 4.5 ml blood samples to measure Xa and rivaroxaban plasma concentrations into 5 ml sodium citrated (3.8%) tubes by venipuncture, 24 h after drug administration (drug concentration valley value).

We estimated rivaroxaban plasma concentrations with the Biophen^®^ Direct Factor Xa Inhibitor (Biophen^®^DiXaI, Hyphen BioMed, Neuville-sur-Oise, France), a calibrated chromogenic anti-Xa assay. The procedure was performed on a STA-R^®^ Evolution analyzer according to the manufacturer’s recommendations, using calibrators from Hyphen BioMed. Commercial rivaroxaban anti-Xa assays have demonstrated good accuracy (bias below 8%) and acceptable precision (inter-laboratory coefficients of variation between 6% and 25%). We applied a procedure for low rivaroxaban plasma concentrations (<50 ng/ml), where we diluted plasma samples 1:8 in buffer and we used low concentration standards (Lessire et al., 2018).

### Genetic Tests for ABCB1 Polymorphisms

We drew blood samples from all patients into EDTA tubes and froze them at −20°C until experiments. We used QIAamp DNA Blood Mini kits (Qiagen, CA, United States) to extract genomic DNA from whole blood. We detected the single nucleotide polymorphisms (SNPs) of *ABCB1* including rs1128503, rs1045642, and rs4148738, according to the allelic discrimination performed on the ABI 3730xL (Applied Biosystems, CA, United States), based on the manufacturer’s instructions.

### Statistical Analysis

The three *ABCB1* SNPs were analyzed using the Chi-square test to verify whether the gene distribution of the enrolled patients conformed to the Hardy–Weinberg equilibrium. According to the Hardy–Weinberg equilibrium, the three *ABCB1* SNPs were constant with a *p*-value > 0.05. ANOVA method was used to evaluate associations between genotypes of *ABCB1* loci (rs1045642, rs1128503, and rs4148738) and drug trough concentrations. In order to rule out the possibility of other factors rather than SNP variants that contributed to the difference of rivaroxaban trough concentrations. The statistical analysis of patients’ gender、age and BMI between different SNP groups were done. The gender differences between groups were analyzed by chi square test. Age and BMI differences between groups were analyzed by *t*-test. The associations between genetic polymorphisms and bleeding events were tested *via* Fisher’s exact test. *p* values < 0.05 were considered as statistically significant. All statistical analyses were performed using the SNP assoc and psych packages of R (version 3.5.2). Graphics were generated *via* the ggplot2 R software package.

## Results

We included 155 Atrial Fibrillation patients in this study, 81 men and 74 women, with an average age of 71.98 ± 10.72 years. The average eGFR was 69.99 ± 22.79 See Table 1.

**TABLE 1:** General data of Rivaroxaban recipients (*n* = 155).

Variables	Total number of patients (*n* = 155)
Men	81 (52.26%)
Women	74 (47.74%)
Age (years)	71.98 ± 10.72
eGFR (ml/min·1.73m^2^)	69.99 ± 22.79
BMI	24.52 ± 3.17
Surface area (m^2^)	1.84 ± 0.17
Atrial fibrillation	155 (100%)
Total	155 (100%)

*ABCB1* genotype distribution results at three loci (rs1045642, rs1128503, and rs4148738). For rs1045642, 22 cases (14.19%) had the TT genotype, 70 (45.16%) had the CT genotype, and 63 (40.65%) had the CC genotype. For rs1128503, 65 cases (41.94%) had the TT genotype, 63 (40.65%) had the CT genotype, and 27 (17.42%) had the CC genotype. For rs4148738, 31cases (20.00%) had the GG genotype, 64 (41.29%) had the AG genotype, and 60 (38.71%) had the AA genotype. Statistical tests showed that the *ABCB1* data distribution conformed to the Hardy–Weinberg equilibrium. See Table 2.

**TABLE 2:** Allelic and genotypic frequencies of *ABCB1* (*n* = 155).

SNP	Genotype	*n* (%)	Allele	*n* (%)	Hardy–Weinberg
rs1045642	TT	22 (14.19)	T	114 (36.77)	0.7309
	CT	70 (45.16)	C	196 (63.23)	
	CC	63 (40.65)			
rs1128503	TT	65 (41.94)	T	193 (62.26)	0.0902
	CT	63 (40.65)	C	117 (37.74)	
	CC	27 (17.42)			
rs4148738	GG	31 (20.00)	G	126 (40.65)	0.0697
	AG	64 (41.29)	A	184 (59.35)	
	AA	60 (38.71)			

SNP, single nucleotide polymorphism.

### Associations Between Genotypes of ABCB1 Loci (rs1045642, rs1128503, and rs4148738) and Drug Trough Concentrations

**FIGURE 1:**
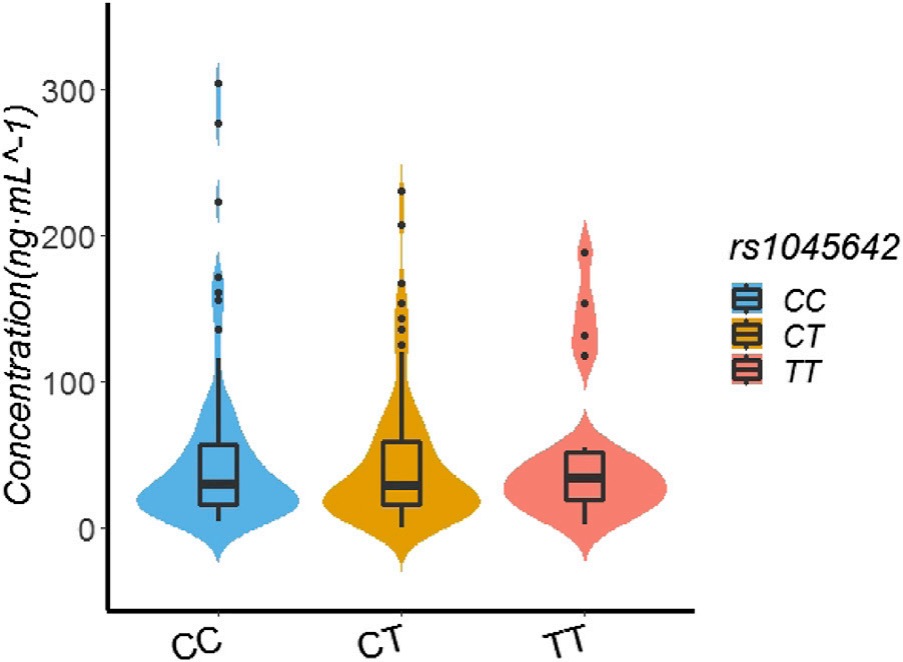
Effect of SNP rs1045642 on Rivaroxaban dose of 155 recipients.

In the patients with the rs1045642 genotype, the rivaroxaban plasma concentration values for those with TT, CT, and CC genotypes were 33.80 ng/ml (19.00, 51.90), 29 ng/ml (15.26, 59.39), and 29.10 ng/ml (15.61, 57.22), respectively. Multiple comparisons between wild (TT) and mutant (CT and CC) genotypes at the rs1045642 locus showed no significant differences of rivaroxaban trough concentrations (TT vs. CT, *p* = 0.586; TT vs. CC, *p* = 0.802; and CT vs. CC, *p* = 0.702). See Table 3; Figure 1.

**TABLE 3:** Effects of rs1045642 SNPs on rivaroxaban plasma concentration in 155 recipients.

Genotype	N	Concentration (ng/ml)
TT	22	33.80 (19.00, 51.90)
CT	70	29.00 (15.26, 59.39)
CC	63	29.10 (15.61, 57.22)

TT genotype compared with CT genotype, *p* = 0.586 > 0.05.

TT genotype compared with CC genotype, *p* = 0.802 > 0.05.

CT genotype compared with CC genotype, *p* = 0.702 > 0.05.

**FIGURE 2:**
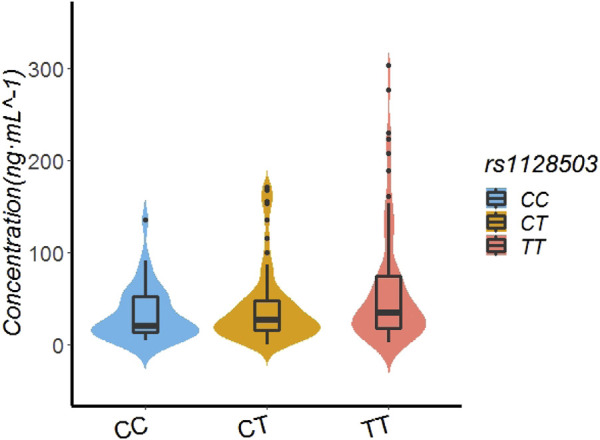
Effect of SNP rs1128503 on Rivaroxaban dose of 155 recipients.

In the patients with the rs1128503 genotype, the rivaroxaban plasma concentration values of those with TT, CT, and CC genotypes were 34.66 ng/ml (18.00.74.00), 26.16 ng/ml (14.88.47.53), and 20.23 ng/ml (13.64.51.70), respectively. Multiple comparisons between wild (TT) and mutant (CC genotypes) at the rs1128503 locus revealed a significant difference of rivaroxaban concentrations (TT vs. CC, *p* = 0.0421). Multiple comparisons between wild (TT) and mutant (CT) genotypes at the rs1128503 locus showed no significant differences of rivaroxaban concentrations (*p* = 0.0651). Multiple comparisons between mutant CT and mutant CC genotypes at the rs1128503 locus showed no significant differences of rivaroxaban trough concentrations (*p* = 0.6127). See Table 4; Figure 2.

**TABLE 4:** Effect of rs1128503 SNPs on rivaroxaban plasma concentrations in 155 recipients.

Genotype	N	Concentration (ng·ml^−1^)
TT	65	34.66 (18.00, 74.00)
CT	63	26.16 (14.88, 47.53)
CC	27	20.23 (13.64, 51.70)

TT genotype compared with CT genotype, *p* = 0.0651 > 0.05.

TT genotype compared with CC genotype, *p* = 0.0421 < 0.05.

CT genotype compared with CC genotype, *p* = 0.6127 > 0.05.

**FIGURE 3:**
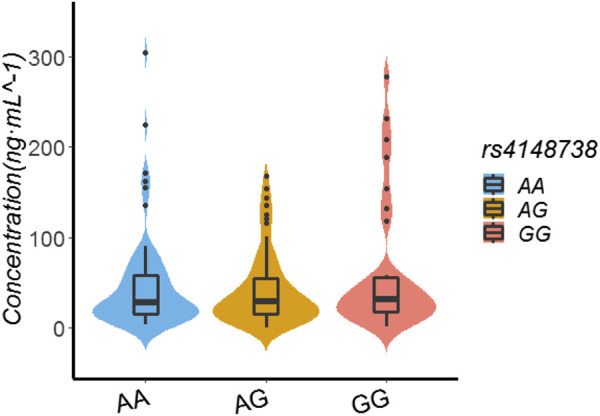
Effect of SNP rs4148738 on Rivaroxaban dose of 155 recipients.

In the patients with the rs4148738 genotype, the rivaroxaban plasma concentration values of those with GG, AG, and AA genotypes were 31.59 ng/ml (17.37.56.11), 29 ng/ml (14.74.54.81), and 28.38 ng/ml (15.80.58.52), respectively. Multiple comparisons between wild (GG) and mutant (AG and AA) genotypes at the rs4148738 locus showed no significant differences of rivaroxaban trough concentrations (GG vs. AG, *p* = 0.341; GG vs. AA, *p* = 0.612; AG vs. AA, *p* = 0.649). See Table 5; Figure 3.

**TABLE 5:** Effect of rs4148738 SNPs on rivaroxaban serum concentrations in 155 recipients.

Genotype	N	Concentration (ng·ml^−1^)
GG	31	31.59 (17.37, 56.11)
AG	64	29.00 (14.74, 54.81)
AA	60	28.38 (15.80, 58.52)

GG genotype compared with AG genotype, *p* = 0.341 > 0.05.

GG genotype compared with AA genotype, *p* = 0.612 > 0.05.

AG genotype compared with AA genotype, *p* = 0.649 > 0.05.

In order to rule out the possibility of other factors rather than SNP variants that contributed to the difference of rivaroxaban trough concentrations. The liver and kidney functions of these 155 patients were all normal. The statistical analysis of patients’ gender, age and BMI between different SNP groups were done. There were no statistical differences between groups. See Table 6.

**TABLE 6:** Main confounding factors between different SNPs groups.

	Age			Gender				BMI		
	No	Mean ± SD	*p*	No	Male	Female	*p*	No	Mean ± SD	*p*
Total	155	71.98 ± 10.70		155				155	24.17 ± 2.87	
Rs1045642										
CC (ref.)	63	71.67 ± 10.57	—	63	33	30	—	63	24.62 ± 2.67	—
CT	70	72.47 ± 10.23	0.658	70	36	34	0.988	70	24.14 ± 3.03	0.336
TT	22	71.32 ± 12.83	0.899	22	12	10	0.861	22	24.02 ± 2.75	0.370
TT vs. CT	—	—	0.666	—	—	—	0.799	—	—	0.869
Rs1128503										
TT (ref.)	65	72.05 ± 10.35	—	65	34	31	—	65	23.79 ± 3.03	—
CT	63	72.98 ± 11.44	0.630	63	30	33	0.596	63	24.47 ± 2.70	0.183
CC	27	69.48 ± 9.68	0.272	27	17	10	0.349	27	24.44 ± 2.87	0.344
CC vs. CT	—	—	0.168	—	—	—	0.182	—	—	0.962
rs4148738										
AA (ref.)	60	71.85 ± 10.42	—	60	31	29	—	60	24.41 ± 2.53	—
AG	64	71.80 ± 10.58	0.978	64	31	33	0.719	64	24.15 ± 2.86	0.594
GG	31	72.61 ± 11.80	0.753	31	19	12	0.382	31	23.83 ± 2.42	0.296
GG vs. AG	—	—	0.735	—	—	—	0.239	—	—	0.593

We summarized the bleeding events of the patients enrolled. Among 155 patients, 24 patients (15.48%) had bleeding events during follow-up. There were two cases of major hemorrhage (1.29%), including one case of cerebral hemorrhage and one case of massive subcutaneous congestion. There is no fatal hemorrhage. The most common bleeding was epistaxis, ecchymosis, gingival hemorrhage and gastrointestinal bleeding. See Table 7.

**TABLE 7:** Description of bleeding events.

Bleeding site	Bleeding events (ratio) Total number of patients (*n* = 155)	Fatal bleeding	Major bleeding	Minor bleeding
Intracranial hemorrhage	1 (0.65%)	0 (0%)	1 (0.65%)	0 (0%)
Epistaxis	8 (5.16%)	0 (0%)	0 (0%)	8 (5.16%)
Gingival bleeding	6 (3.87%)	0 (0%)	0 (0%)	6 (3.87%)
Ecchymosis	7 (4.52%)	0 (0%)	1 (0.65%)	6 (3.87%)
Gastrointestinal bleeding	2 (1.29%)	0 (0%)	0 (0%)	2 (1.29%)
Total	24 (15.48%)	0 (0%)	2 (1.29%)	22 (14.19%)

We analyzed possible associations between bleeding events and *ABCB1* gene polymorphisms. No significant correlation between rs1045642, rs1128503, and rs4148738 locus variations and the occurrence of bleeding events were obtained (*p* > 0.05). Table 8 shows the results.

**TABLE 8:** *ABCB1* SNPs and bleeding events.

SNP	Genotype	Hemorrhage	No-hemorrhage	*p*
rs1045642	TT	3	19	0.9107
	CT	10	60	
	CC	11	52	
rs1128503	TT	9	56	0.8062
	CT	10	53	
	CC	5	22	
rs4148738	GG	3	28	0.5979
	AG	10	54	
	AA	11	49	

## Discussion

The influence of genetic factors on drug responses is complex, and genetic structure differences are thought to be the main factors leading to individual differences (Paré et al., 2013; Dimatteo et al., 2016; Rodríguez-Vicente et al., 2016). Genetic polymorphisms in a series of metabolic enzymes, transporters, receptors, and other drug targets involved in the process of drug delivery *in vivo* cause individual differences in drug efficacy and toxicity (Klein et al., 2017; Tseng et al., 2018). P-gp is an important transporter for many drugs *in vivo* (Choo et al., 2000).

The activity and expression of P-gp can affect the absorption, distribution, metabolism, and excretion of drugs. Studies have shown that *ABCB1* gene polymorphisms may cause changes in its activity and expression, resulting in individual differences in drug efficacy and adverse reactions. Rivaroxaban is the metabolic substrate of P-gp. *ABCB1* gene polymorphisms may affect the dose-effect relationship and the bleeding risk of rivaroxaban.

There are some studies on the polymorphism of *ABCB1* gene encoding P-gp, but only few clinical studies have evaluated whether they explain the difference in pharmacokinetics of rivaroxaban (Asic et al., 2018). At present, no clinically significant *ABCB1* SNPs have been identified for rivaroxaban. The evidence for the clinical application of individual therapy under the guidance of rivaroxaban pharmacogenomics is lacking. Current evidence levels are mostly based on case reports (Ing Lorenzini et al., 2016) and a small pharmacokinetic cohort study (Gouin-Thibault et al., 2017). An animal study of genetically altered mice lacking both *ABCB1* and *ABCG2* demonstrated significant serum peak concentration increases due to reduced rivaroxaban clearance (Gong et al., 2013). Additionally, a pharmacokinetic study on healthy volunteers with different *ABCB1* genotypes (SNPs rs2032582 and rs1045642) showed that *ABCB1* genotypes were not a significant determinant of interindividual variability in terms of serum drug concentrations (Gouin-Thibault et al., 2017). The changes in rivaroxaban blood concentrations affect the drug’s effects and may lead to embolism or hemorrhage events. However, only one case report has suggested that defects in *ABCB1* may increase the risk of hemorrhage (Ing Lorenzini et al., 2016).

There are also *in vitro* studies on it. This study (Sennesael et al., 2018) confirmed that no association between *ABCB1* genotype and intracellular accumulation of rivaroxaban. However, same study which is done in human embryonic kidney cells HEK 293 confirmed the correlation between the increased *ABCB1* expression and decreased intracellular rivaroxaban accumulation. It can be seen that there is little research on this aspect, and the conclusions of existing studies are not completely consistent.

Me anwhile, not only the correlation between *ABCB1* genotype and accumulation of rivaroxaban but the correlation between gene polymorphism and P-glycoprotein expression has not been unified. Existing studies have shown that *ABCB1* gene C3435T, G2677T/A, C1236T and T-129C polymorphisms are associated with the expression of P-glycoprotein and mRNA, but the expression of protein and mRNA related to gene polymorphism is inconsistent. The reason may be different splicing patterns of mRNA. Because the same gene can have different transcripts, it can express many proteins with different structures; It is also possible that during the process of mRNA translation into protein, mRNA will degrade or stop translation, resulting in the increase of mRNA level but the decrease of protein expression; The difference of half-life between mRNA and protein may also lead to inconsistent expression of mRNA and protein; There may be linkage between *ABCB1* SNPs; It may be affected by physiological factors (such as endogenous factors: hormone levels) and pathological factors; Different tissue types and experimental methods selected in different studies will also lead to different results, which will affect the stability, processing and expression of protein and mRNA.

So, at present, the correlation between gene polymorphism and P-glycoprotein expression has not been unified. Moreover, there are great differences among different races. Most of the existing data are obtained from Caucasian people, while the data of Chinese people are very few.

Therefore, we need further study to explore the correlation between *ABCB1* gene polymorphism and P-glycoprotein expression. In particularly, more systematic research on Chinese population in terms of multi organization, multi-location, pharmacokinetics and pharmacodynamics should be carried out. And further study on the *ABCB1* gene polymorphism and P-glycoprotein expression and function differences on the impact of drug disposal is useful to provide theoretical and practical basis for clinical rational drug use.

Our study suggests that the presence of the rs1128503 locus of the *ABCB1* gene is correlated with the valley concentration of rivaroxaban in individuals of Mongolian descent, but rs1045642 and rs4148738 have no associations with serum drug concentrations. But no significant correlation between locus variations and bleeding events were obtained. This is the first study to explore the effect of *ABCB1* SNP locus on rivaroxaban concentration and bleeding events in Chinese population. It is also the first study to find rs1128503 locus associated with rivaroxaban valley concentration. Therefore, it has clinical significance and value. At the same time, some special cases in the study are very enlightening to us. A patient we followed in this study, we found that testing the serum drug concentration repeatedly resulted in a 10-fold difference between the two steady state valley serum concentrations. We excluded data of this patient from our statistical analysis, but we actively sought out the underlying causes and found that the patient was using an antibiotic known to inhibit the *ABCB1* product the second time we tested the drug concentration, leading to the dramatic concentration increase. The patient was taking “fluconazole” when the second time we tested the concentration of rivaroxaban. And, the rs1128503 is variant in this patient. When the patient stopped the antibiotic, the serum concentration of rivaroxaban dropped to its initial value. Co-administration of a P-gp inhibitor with rivaroxaban may warrant caution in patients at risk of overexposure. Drug interactions and genetic variation play a role in this variation. Thus, dosing for rivaroxaban depending on genotyping results remains a possibility. Large population studies are required to clarify the clinical significance of genotyping for this drug.

No significant correlation between the presence of *ABCB1* gene polymorphisms and the occurrence of hemorrhage events were obtained. But we are aware of the small sample size of our study, and associations between gene polymorphisms and hemorrhage events need to be further verified by in large sample size studies. We will continue to follow patients up to gather data for future studies. And another thing is that we only tested the DNA, the expression level of *ABCB1* gene was not determined including mRNA and protein. Quantitative analysis of *ABCB1* will be done in our future study which will be meaningful.

The DOACs have been used with increased frequency during the past 2 years in China, and their related pharmacogenomics research is small, and large population studies are needed to explore the role of genes in anticoagulant therapy. With the progress of pharmacogenomics and the gradual elucidation of the molecular mechanism of anticoagulant agents, genes will be found in different ethnic and regional populations with the development of genome-wide association studies. With the application of exon and genome-wide sequencing technologies, new genes and mutation sites will be identified (Scott et al., 2012), and individual pharmacogenomics will be accurate and rational for patients on anticoagulant therapy, and individualized treatment will become a reality.

## Conclusion

This study suggests that the *ABCB1* gene rs1128503 variant is correlated with the valley concentration of rivaroxaban in individuals of Mongolian descent. But no significant correlation between rs1128503 locus variations and bleeding events were obtained.The association between gene polymorphisms and hemorrhage events needs to be further verified by expanding the sample size. Co-administration of a P-gp inhibitor with rivaroxaban may warrant caution in patients at risk of overexposure. Depending on genotyping results for rivaroxaban dosing in clinical practice remain open for NOACs. Large population studies are required to clarify the clinical significance of genotyping for this drug class.

## Data Availability

The raw data supporting the conclusions of this article will be made available by the authors, without undue reservation, to any qualified researcher.
